# Ophthalmology

**Published:** 1890-12

**Authors:** Alvin A. Hubbell

**Affiliations:** Buffalo, N. Y.


### OPHTHALMOLOGY.

CONDUCTED BY
ALVIN A. HUBBELL, M. D., Buffalo, N. Y.

BORACIC ACID WITH MASSAGE IN DISEASES OF THE CONJUNCTIVA.—Dr. W. M. Beaumont recommends rubbing the conjunctiva with finely powdered boracic acid in granular and follicular conjunctivitis. He everts the upper lid in the usual way, and sprinkling the exposed conjunctiva thickly with the powder he rubs it into it for five or ten seconds with the end of the index finger. The acid then remaining may be washed away with a soft brush dipped into "lead lotion," or if there is not much discomfort it is allowed to remain. The lower lid is then treated in the same way. At first the applications are somewhat painful, but afterwards less so. Pain may be prevented by using cocaine beforehand. The treatment may be repeated every day, or every two days. After two or three applications the conjunctiva presents a healthy reaction.—*Lancet*, October 18, 1890.

PILOCARPINE IN OCULAR THERAPEUTICS.—Dr. G. Staderini, after making many experiments with pilocarpine, has arrived at the following conclusions: Subcutaneous injections of gr. $\frac{1}{10}$ to $\frac{1}{8}$ of the nitrate of pilocarpine are useful in several inflammatory diseases of the eyes, and especially in those that are the consequence of rheumatism, as episcleritis, iritis, and iodiophathic optic neuritis. They subdue inflammatory conditions of the iris and of the ciliary body which supervene when masses of the cortical substance of the lens remain in the anterior chamber after the operation of extraction of cataract. Further, they facilitate the absorption of these residual fragments; and they promote the absorption of non-organized opacities in the vitreous humor, especially when these opacities are the consequence of recent infiltration. The last-named effect is the most valuable of all the actions of pilocarpine. Improvement of vision also takes place after pilocarpine injections in cases of progressive myopia, and in cases of detachment of the retina, though the effects here are only transient. Lastly, they may be employed with good effect in certain forms of amblyopia, and of amaurosis of unknown origin, but which are probably the consequence of disorder of the circulation followed by serious transudation in the sensory nerve centres.— *Annali di Otalmologia. Therapeutic Analyst.*

---

Treatment of Trachoma with Bichloride of Mercury.—Drs. Gust and Otto Keining (*Deutsch. Med. Wochen.*, Oct. 9, 1890,) advocate a method which they say they have found so efficient that cases which have been under treatment by other means for long periods have been cured in from two to six weeks, even when there has been extensive pannus. The eye is first thoroughly irrigated with corrosive sublimate, 1 to 3,000, and then the lids are everted and firmly rubbed with a hard pad soaked in the same solution. If the ocular conjunctiva is affected, it is treated in the same way, any bleeding from granulations being disregarded. The friction employed is proportionate to the severity of the case. When the granulations are so firm that the friction does not evacuate their contents, they are torn open with toothed forceps, or emptied by being squeezed with cilia forceps. The treatment is repeated daily. Some reaction follows, but this need not prevent a repetition of the process. There is always rather free secretion at first, but this disappears after the third to the fifth day of treatment. To remove the secretion, the eyes are bathed for an hour three times a day in a warm solution of the bichloride, 1 to 10,000, and the same plan is employed whenever there is itching of the lids. If the swelling of the lids becomes considerable, the treatment is discontinued, and the lids are brushed with the milder antiseptic. Sometimes at the commencement of the treatment membranes form on the conjunctiva; these should be allowed to come away spontaneously, and the conjunctiva touched only where it is free from them.— *British Medical Journal*, Supplement, October 25, 1890.

---

Græfe's Lid-Sign in Exophthalmic Goitre.— Dr. Sharkey, at a recent meeting of the Ophthalmological Society of the United Kingdom, (October 16, 1890,) presented the results of investigations on this subject which he had made upon 613 cases. Græfe described this sign in 1864 as an absence of correspondence between the movement of the lids and the elevation and lowering of the visual planes, and considered it pathognomonic of exophthalmic goitre. Sharkey's cases included diseases of all kinds other than exophthalmic goitre, and out of the 613 cases, twelve, or a little less than two per cent., presented the sign well marked. Many others showed it so long as they stared at the object held before them. "A large proportion of healthy people can voluntarily produce the lid-sign in themselves by staring." Inasmuch, then, as Græfe's lid-sign is far from always being present in undoubted cases of exophthalmic goitre, and is often very well marked in others who certainly have not this disease, it cannot be considered very valuable as a diagnostic sign.

